# Electrical Interconnection and Bonding by Nano-Locking

**DOI:** 10.3390/nano11061589

**Published:** 2021-06-17

**Authors:** Jielin Guo, Yu-Chou Shih, Frank G. Shi

**Affiliations:** 1 Department of Materials and Manufacturing Technology, Henry Samueli School of Engineering, University of California, Irvine, CA 92617, USA; 2 Department of Chemical and Biomolecular Engineering, Henry Samueli School of Engineering, University of California, Irvine, CA 92617, USA; yuchous@uci.edu (Y.-C.S.); fgshi@uci.edu (F.G.S.)

**Keywords:** nano-locking, die attachment, heterogeneous integration, electrical contact resistance, junction temperature, lumen output, wet high temperature operating life (WHTOL), flip-chip LED

## Abstract

The growing demand for increased chip performance and stable reliability calls for the development of novel off-chip interconnection and bonding methods that can process good electrical, thermal, and mechanical performance simultaneously as well as superior reliability. A chip bonding method with the concept of “nano-locking” (NL) is proposed: the two surfaces are locked together for electrical interconnection, and the connection is stabilized by a dielectric adhesive filled into nanoscale valleys on the interconnecting surfaces. The general applicability of this new method was investigated by applying the method to the die-substrate bonding of two different packages from two different manufacturers. Electrical, optical, and thermal performances as well as reliability tests were carried out. The surface morphology of the bonding package substrates plays an important role in determining the contact resistance at the bonding interfaces. It was shown that samples with different roughness height distribution on the metallic surfaces formed a different total number of contacts and the contact area between the two bonding surfaces under the same bond-line thickness (BLT): a larger number of contact area resulted in a reduced electrical resistance, and thus an improved overall device performance and reliability.

## 1. Introduction

To fulfill the next-generation electronic device performance requirements, advanced packaging must press for innovations in process, materials, and equipment. Heterogeneous integration is proposed to build large systems out of smaller functions and enables semiconductor dies with different feature sizes integrated into a single package. Advanced 3D integration and packaging methods including through silicon via (TSV), ball grid area (BGA), micro pillar grid area (MPGA), intel’s embedded multi-die interconnect bridge (EMB), etc. provide the solutions to the vertically stacking integrated circuits (ICs) [1]. A typical chip-to-package electrical interconnect/bonding often utilizes electrically conductive die attach adhesives (DAAs), die attach films (DAFs), or solders to secure a reliable thermal and electrical conduction path between the chip and substrate [2]. In conventional manufacturing, for a typical chip-to-package electrical bonding using a liquid DAA to be reliable, its bond-line thickness (BLT) is often required to be as thick as over 25 μm, while the newly developed DAF can reduce the BLT to as thin as 10 μm. For chip-to-package electrical interconnections using a solder, a typical BLT thickness of the order of 30 μm is required to avoid thermo-mechanical reliability problems [3]. Indeed, the interconnection and bonding technologies have become the bottleneck to achieving high integration density while keeping the enhanced device performance and high yield [4]. The current two mainstream electrical interconnection and bonding methods including bump and bumpless-based approaches are facing the challenges of further shrinkage of the bump size and interconnect pitch size due to the physical limits [5,6]. The decreasing travel distance for signals is important to reduce the power consumption, latency, and heat generation while improving the device performance [7,8].

To better solve the challenges above-mentioned, an innovative die-substrate bonding method is proposed with a concept of “nano-locking” (NL): the two surfaces are locked together for electrical interconnection, and the connection is stabilized by a dielectric adhesive filled into nanoscale valleys on the interconnecting surfaces. Compared with the traditional DAAs, no metallic fillers are added inside the dielectric adhesive. Besides, no additional time-consuming and expensive pre-annealing plasma activation in an ultra-high vacuum is needed in order to obtain a super-flat surface to achieve further precise alignment during the bonding process.

When the two surfaces are brought into contact under bonding pressure, surface roughness causes contact to occur at discrete contact spots. The sum of the areas of each contact spot constitutes the real overall contact area [9]. The real overall contact area of the present NL bonding method is a function of the surface morphology of the metallic pads and the pressure applied on the bonding interfaces. The real contact area in turn influences the transmission of the heat and electric current across the contact interface. The multiscale roughness of surfaces has been traditionally characterized by statistical parameters [10]. Therefore, the study of the surface morphology on metallic pads is very important in determining the real overall contact area, which will in turn lead to an effect on the transmission of the electric current and heat across the contact interface [11].

The objective of the present work was to demonstrate the potential of an innovation for establishing a simultaneous mechanical, thermal, and electrical connection between two metallic surfaces without requiring a prior time-consuming and expensive surface nanoscopic planarization and without requiring any intermediate conductive material. Furthermore, this study also focused on the influence on the contact resistance brought by different surface morphologies on the metallic pads. By analyzing the surface height distribution of metallic pads on semiconductor die and package substrates, the formation of the total contact number and the real overall contact area can be evaluated. Only the real overall contact area contributes to the electrical and thermal conduction between the bonding surfaces and will change the electrical (and thermal) contact resistance of the overall NL bonding layer. Two different package substrates from different manufacturers were employed to make a comparison when applied to the die-substrate bonding of the same semiconductor dies with the same bond-line thickness (BLT). A mathematical model is proposed to describe and predict the electrical (and thermal) conductivity of the overall real contact area between the bonding surfaces. The electrical, thermal, and optical performance evaluation of the packaged devices with the NL bonding method using different package substrates were carried out and compared respectively. The larger effective overall contact area leads to smaller contact resistance and contributes to the improvement in the device performance and reliability.

The study of the effect of surface morphology on the NL bonding methods is of high importance since it exhibits a profound effect on the overall response of the electronic device subject to various device performance and reliability.

## 2. Experiments

**Figure 1:**
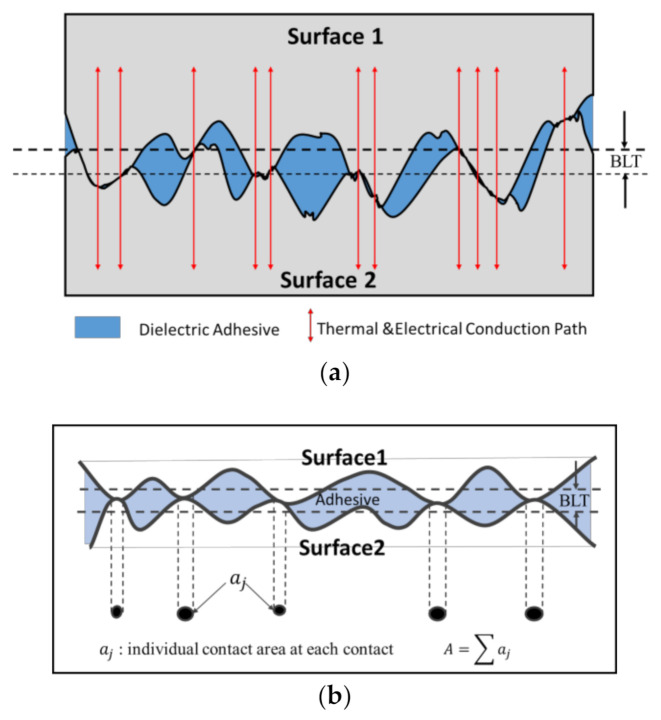
(**a**) Schematic drawing of “nano-locking” bonding between two surfaces with random roughness with the assistance of a dielectric adhesive between two surfaces. (**b**) Illustration of overall real contact area (A) between the two bonding surfaces dependent on the intrinsic nanoscopic structures and contact at each spot: the overall contact area (A ) is the sum of each individual contact area ($a_{j}$ ).

The concept of “nano-locking” (NL) refers to the interconnection between the random intrinsic nanoscopic structures on the two metallic surfaces when they are brought together, and such an interconnection can be mechanically stabilized and bonded by filling the nanoscopic valleys with a dielectric adhesive. As the two metallic surfaces are brought into contact, nanoscale asperities are the first to come into contact, merging to form contacts to establish the electrical and thermal conduction, as shown in in Figure 1a [12]. The bond-line thickness (BLT) is defined as the vertical distance between the baseline of the surface roughness on the two contacted bonding surfaces, as shown in Figure 1a [13]. The potential range of BLT is within the maximum and minimum limits, controlled by the highest ridges and deepest valleys on the interconnecting surfaces.

The real contact area between the two bonding surfaces is the sum of the individual nanoscopic “asperities” at each contact spot, as shown in Figure 1b. Therefore, the surface morphology plays an important role in determining the real contact area, which in turn will lead to an effect on the transmission of electric current and heat across the contact interface.

### 2.1. Fabrication of Packaged Devices

The NL bonding method has been successfully demonstrated by bonding high-power GaN based flip-chip dies to the device substrate. The as-received commercially available semiconductor dies used in this study were flip-chip type light emitting diodes (LEDs) with the size of $1\times 1{\;\mathrm{mm}}^{2}$ and a forward voltage of 3.0 V (Lextar Electronics Corp., Hsinchu, Taiwan). The composition of the die pads consisted of Ti/Ni/Au. Two types of substrates with different surface morphology (type-I and type-II) were used for the die-substrate packaging. The package substrate had a size of $5\times 6{\;\mathrm{mm}}^{2}$ and consisted of an optically reflective cup and heatsink slug (Lextar Electronics Corp., Taipei, Taiwan and Jufei Optoelectronics Corp., Shenzhen, China). The composition of the substrate pad was Cu/Ni/Ag.

**Figure 2:**
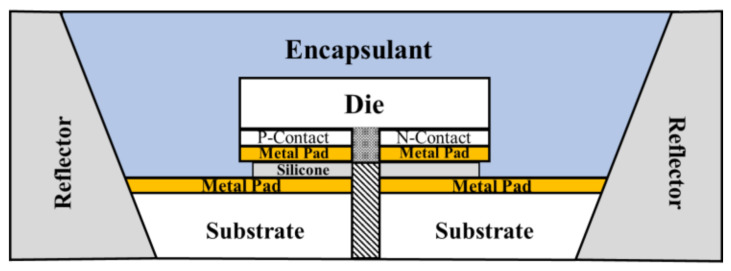
Cross-sectional view of a packaged flip-chip type high-power GaN based die via the NL bonding method.

The semiconductor die bonder employed was a conventional Mech-EI manual die bonder. A commercially available liquid dielectric adhesive was used to fill the valleys on the interconnecting surfaces, and then subsequently cured in an oven at a temperature of 150 °C for one and half hours. The encapsulation of the die-package was through a commercially available silicone resin (Dow Inc., Midland, MI, USA, and the encapsulation was completed by another thermal curing at 150 °C for two hours. The packaged device was then soldered to an Al-based printed-circuit-board before performing any property measurements. The BLT value of the two sets of devices was the same, which was controlled around 100 ± 5 nm. Figure 2 shows the cross-sectional view of the whole package.

For the purpose of reference and comparison, the devices made by commercially available die-substrate bonding methods were prepared, as shown in Table 1.

### 2.2. Devices Performance Evaluation

The surface roughness of metallic pads on the die and substrate was determined by atomic force microscopy (Anton Paar Tosca 400 AFM, Graz, Austria) using an Arrow NCR cantilever with a reflective aluminum coating that has a typical tip radius of <10 nm, resonance frequency of 285 kHz, and spring constant of 42 N/m. Images were acquired using a scan rate of 1 line/s and measurement region of 50 × 50 um^2^. The BLT was observed by scanning electron microscope (SEM, Tescan GAIA-3 GMH, Brno, Czechia) of the cross-sectional samples prepared by focus ion beam (FIB). The current-voltage (I−V) behavior of the fabricated device was measured and recorded by using the Keithley 2450 source meter (Cleveland, OH, USA). The junction temperature was measured as a function of time by following the diode forward method and thermal resistance of the overall die-substrate interconnection, and the bonding layer was then calculated [14]. The current source with an input constant current of 700 mA was supplied by an Everfine power generator (Hangzhou, China), and the lumen output was measured in a LabSphere integral sphere (North Sutton, NH, USA). The wet high temperature operation life (WHTOL) reliability test was carried out in a chamber (GLMP50, Chemkorea Corp., Irvine, CA, USA) where the temperature and humidity could be controlled under biased condition. All the packaged devices were placed on the heat sink in the case of overheating and the lumen maintenance was evaluated as a function of aging time at a high temperature of 85 °C and a relative high humidity of 85% with the maximum suggested input DC current of 700 mA, which was aged beyond the requirement of the industrial standard JEDEC No.22-A101C by extending the test duration by 25% from 1000 h to 1250 h.

## 3. Results and Discussion

### 3.1. Surface Morphology Study

**Figure 3:**
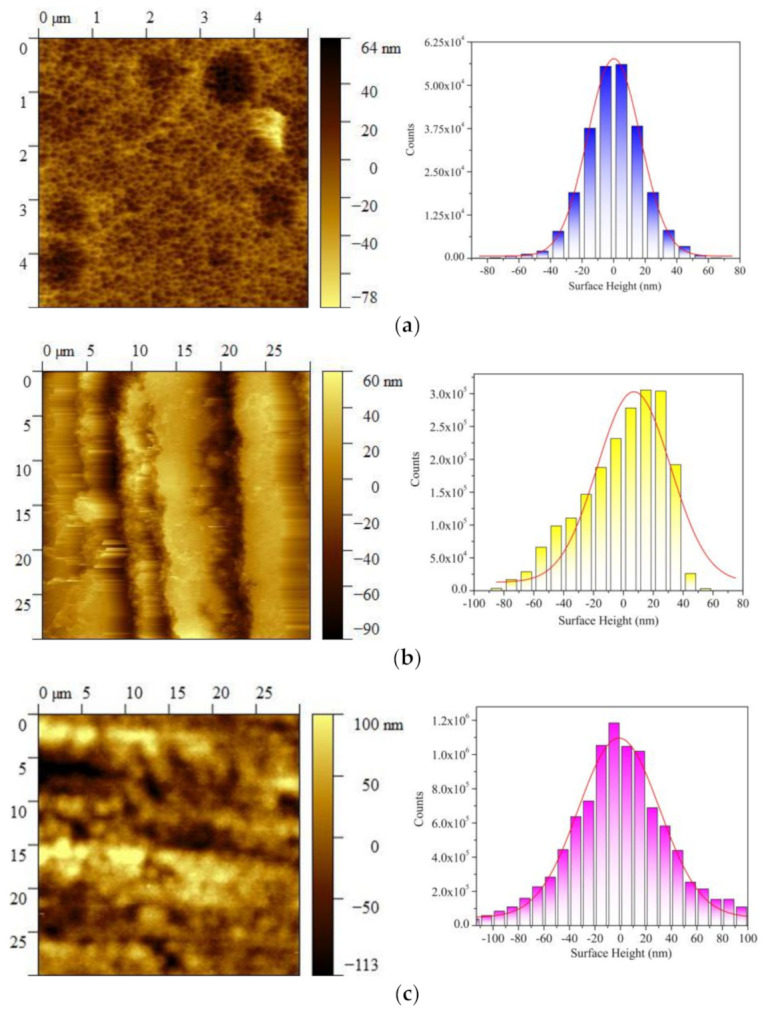
Atomic force microscopy (AFM) for the surface topography and surface roughness distribution. (**a**) The semiconductor die metallic pads; (**b**) the package substrate-I metallic pad; (**c**) the package substrate-II metallic pad.

Figure 3 presents the surface topography and the histogram of roughness height distribution for the metallic pads on the die and substrate. As shown in Figure 3a, the surface roughness of the semiconductor die ranged from −(78 ± 2) nm to +(64 ± 2) nm. Figure 3b,c presents the AFM image as well as the surface roughness distribution of the different two types of substrate metallic pads. It was noted that the surface roughness of substrate-I ranged from −(90 ± 2) nm to +(60 ± 2) nm, and the surface roughness of substrate-II ranged from −(113 ± 2) nm to +(100 ± 2) nm.

**Figure 4:**
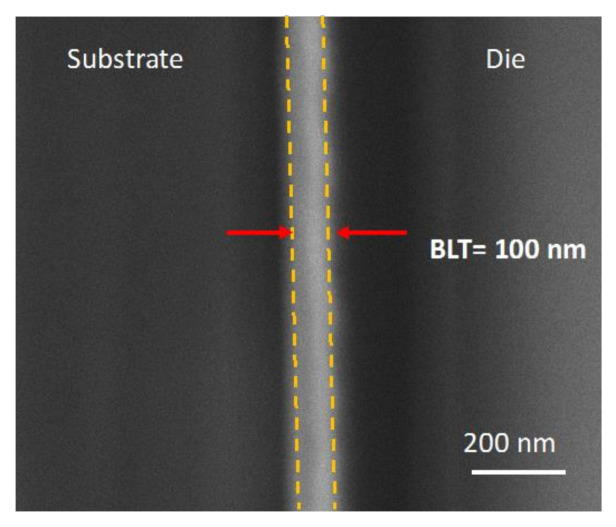
SEM/FIB observation of the BLT of the nano-locking chip bonding approach.

The cross-sectional SEM images were taken at various different locations along the die–substrate bonding layer for each sample and the mean was taken to estimate the BLT value, marked with yellow dashed lines in Figure 4. Most of the heights of the peaks and valleys on the metallic pads of die and substrate were between 0–20 nm according to the AFM measurements (see Figure 3). Therefore, the boundary of the BLT looked quite flat in the SEM images.

The contact electrical (and thermal) conductivity based on the formation of the “nano-locking” structure at each contact spot can be summarized as a function of surface height distribution:

(1) $$\gamma =\alpha \;\cdot \;\left[w_{1}r_{1}+w_{2}r_{2}+\ldots +w_{i}r_{i}\right],$$

where $w_{i}$ is the probability of the contacts for the certain surface heights range; $r_{i}$ is the ratio of the certain surface heights range; n is the number of the bins considered for the surface height distribution that establishes the effective “nano-locking” structure under certain BLT; and α is the conductivity of the unit effect area.

The $w_{i}$ is a binary value considered by the threshold method for simplification [15]. When the certain height distribution of the surface roughness contributes to the formation of the “nano-locking” structure under specific BLT,$\;w_{i}$ = 1, otherwise, if the certain height distribution does not contribute to the formation of the “nano-locking” structure, $w_{i}=0$. In the real-world scenario, the $w_{i}$ should be a continuous value between 0 and 1. Then, the value of $w_{i}$ can be further studied by using machine learning, which was out of the scope of this study [16].

To achieve the electrical interconnection with specific BLT = 100 ± 5 nm, the surface roughness height range of the package substrate was selected according to the surface roughness height distribution on the semiconductor die to establish the effective “nano-locking” structure. The “frequency percent” of the surface roughness height distribution on the bonding surface is defined as: the number of heights that belong to a certain qualified range/total number of heights in the same scanning unit area. Table 2 describes the certain frequency percent distribution of the surface roughness heights for the die and the two different types of package substrates. The total frequency percent of qualified surface roughness height distribution for substrate type I (SUB-I) was about 11% and the total frequency percent of the qualified surface roughness height distribution for substrate type II (SUB-II) was about 21%.

**Figure 5:**
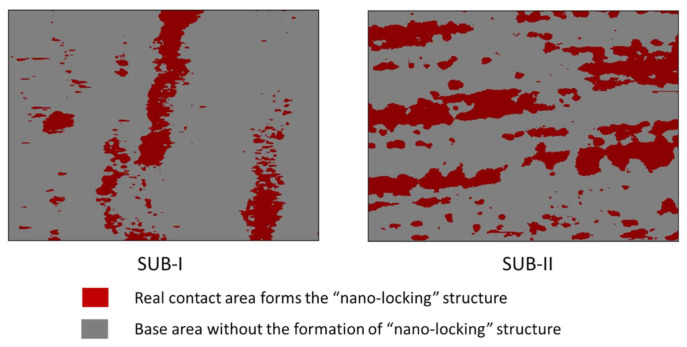
Real contact area distribution on the metallic pads of the two different types of package substrates under the same BLT.

Since the BLTs of the two packages were controlled the same, the surface morphology of the metallic pads on semiconductor die was the same, therefore, the overall bonding layer using the substrate type II (SUB-II) had a larger total contact number and contact area compared with the case using the substrate type I (SUB-I). The real contact areas are defined as when the ridges (positive surface height) and valleys (negative surface height) on the surface roughness of two bonding surfaces make contact with each other and form the electrical/thermal conduction within the desired BLT (BLT = 100 ± 5 nm in this case). Based on the AFM analysis shown in Figure 3, each ridge/valley on the scanning area of the surface has a responding height and their location can be described with unique coordinate. Then, the coordinate of the whole effective area can be acquired and plotted by using the “contour” function by Matplotlib, as shown in Figure 5. Figure 5 shows the distribution of the real effective contact area (red area) on the two different package substrates within the scanning area, which forms the “nano-locking” structure under the same BLT.

### 3.2. Devices Performance: Electrical

**Figure 6:**
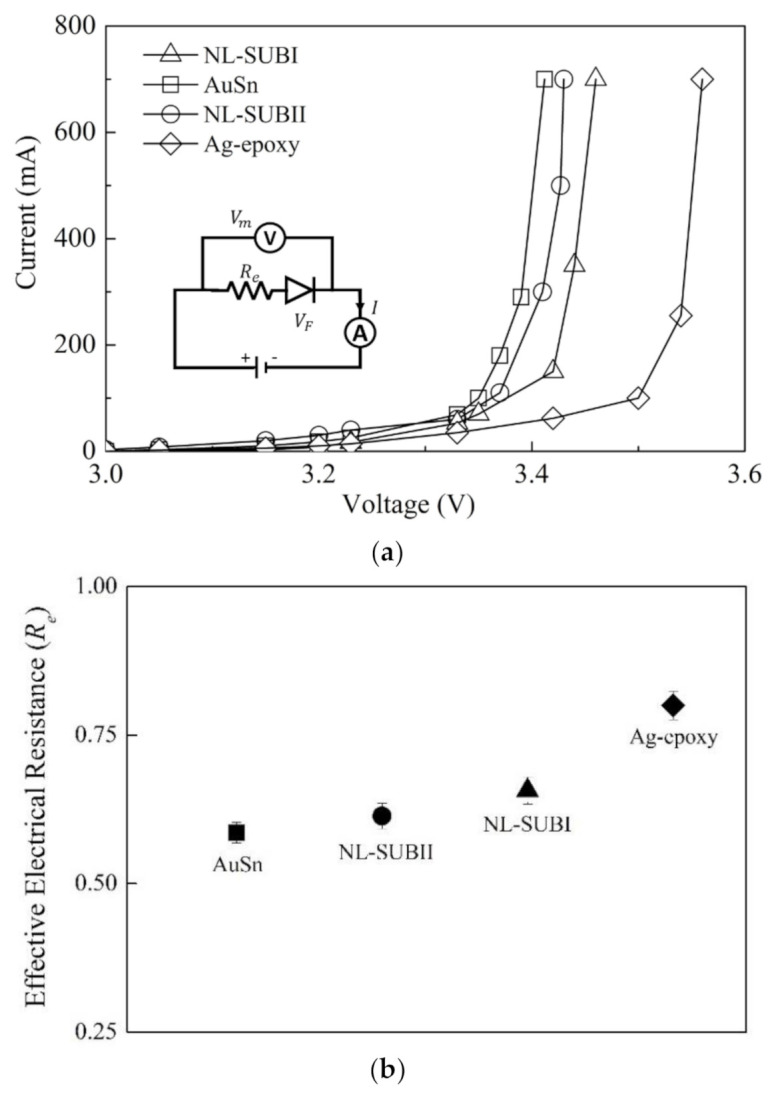
(**a**) Measurement of the current (I) and voltage (V) relationship for the devices made of the present NL with different substrates and conventional die–substrate interconnection and bonding methods. The open square symbol (□) represents the AuSn bonding; the open circle symbol (◯) represents the NL bonding with substrate type-II; the open triangle symbol (△) represents the NL bonding with substrate type-I; the open rhombus symbol (◇) represents the Ag-epoxy bonding. The solid curve represents the best I-V fitting. (**b**) The $R_{e}$ of packaged devices made by NL bonding with different substrates and different die–substrate interconnection and bonding methods: the solid square symbol (■) represents AuSn bonding with an industrial standard BLT value of 20 ± 2 μm; the solid circle symbol (●) represents the NL bonding with substrate type-II; the solid triangle symbol (▲) represents the NL bonding with substrate type-I; the solid rhombus symbol (◆) represents the Ag-epoxy bonding with an industrial standard BLT value of 25 ± 2 μm.

Figure 6a presents the electrical performance for devices made by the NL bonding method with different package substrates and its comparison to two conventional die-substrate bonding methods. According to Figure 6a, the measured voltage for the devices made by the NL approach were all smaller than that for the devices using the Ag-epoxy bonding method and larger than that for the devices using the AuSn bonding method under the same forward current of 700 mA. The effective interconnection electrical resistance $R_{e}=dV/dI=\left(\;V_{m}-V_{F}\right)/I_{F}$, where $V_{m}$ is the measured device voltage, $V_{F}$ is the forward voltage, and $I_{F}$ is the forward current.

Figure 6b presents the extracted $R_{e}$ value of devices made by the NL bonding method with different substrates and their comparison to two conventional die-substrate bonding methods. The $R_{e}$ of devices made by NL bonding with package substrate type I (SUB-I) was about 7% higher than the $R_{e}$ of NL bonding with package substrate type II (SUB-II).

This is easily understandable because the package substrate type II has a larger frequency percent of the qualified surface height distribution that can establish a larger total number of contacts, resulting in a larger overall contact area under the same BLT, as shown in Table 2. For the NL bonding case, the real contact area can be considered as the sum of the contact areas at each contact spot [17]. For nanoscale contacts, it has been shown that geometry conditions affect the real contact area differently [18]. Therefore, the $R_{e}$ of NL bonding with package substrate type II (SUB-II) is decreased.

In addition, the$\;R_{e}$ value of the NL bonding method with package substrate type II (SUB-II) was about 4.8% larger than in the case of AuSn bonding and was about 23% smaller than the case of Ag-epoxy bonding. This is because defects such as voids, delamination, or cracks introduced at the interfaces during the curing process for Ag-epoxy will largely increase the interfacial resistance and degrade the corresponding electrical performance [19,20].

### 3.3. Device Performance: Thermal

**Figure 7:**
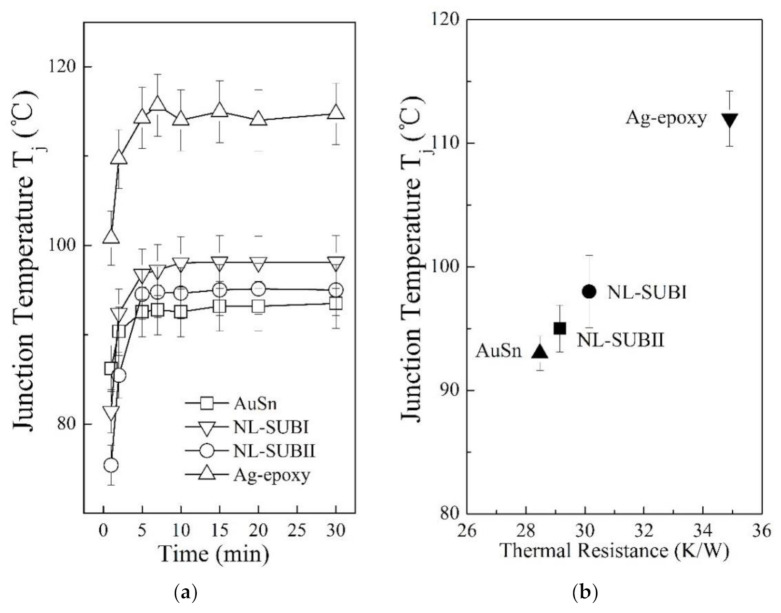
(**a**) The die junction temperature ($T_{j}$) of the devices made by the NL bonding method with different substrates and two conventional die–substrate bonding methods: the open circle symbol (◯) represents the $T_{j}$ data for the device made by NL bonding with substrate type-II, the open downside triangle symbol (▽) represents $T_{j}$ data for the device made by NL bonding with substrate type-I, the open square symbol (□) represents $T_{j}$ data for the device made by the AuSn bonding with an industrial standard BLT value of 20 ± 2 μm, the open upside triangle symbol (△) represents Ag-epoxy bonding with an industrial standard BLT value of 25 ± 2 μm. (**b**) The relationship between $T_{j}$ and $R_{th}$: the solid circle symbol (●) represents NL bonding with substrate type-I; the solid square symbol (■) represents NL bonding with substrate type-II; the solid upside triangle (▲) represents AuSn bonding with an industrial standard BLT value of 20 ± 2 μm; and the solid downside triangle (▼) represents Ag-epoxy bonding with an industrial standard BLT value of 25 ± 2 μm.

Figure 7 presents the thermal performance for the devices made by the NL approach with different substrates and two conventional die–substrate bonding methods. Figure 7a presents the measurements of the die junction temperature ($T_{j})$ of packaged devices. It is evident that the device made by the NL bonding method with substrate type I (SUB-I) was about 3 °C higher than the devices made by substrate type II (SUB-II). In addition, the junction temperature ($T_{j})$ of devices using the NL bonding method with substrate type II (SUB-II) was about 2 °C higher than that of the AuSn bonding, and about 19 °C lower than the Ag-epoxy bonding.

In Figure 7b, the thermal resistance$\;\left(R_{th}\right)$ of the packaged devices is proportional to its junction temperature ($T_{j})$. The $R_{th}$ of the packaged devices using NL with substrate type II (SUB-II) was about 3% smaller than the case of using substrate type I (SUB-I), and was about 2% larger than the AuSn bonding approach, and about 16.5% smaller than the Ag-epoxy bonding approach.

As discussed above, the package substrate type II has a larger frequency percent of the qualified surface heights distribution, which can establish a more effective total number of contacts and larger contact area under the same BLT, as shown in Table 2. Therefore, the $R_{th}$ of devices made by package substrate type II will be decreased. However, for the case of Ag-epoxy bonding, the existence of defects at interfaces during the curing process can greatly affect the interfacial resistance and heat dissipation and lead to a large increase for the overall $R_{th}$, which degrades the thermal performance.

### 3.4. Devices Performance Evaluation: Optical

**Figure 8:**
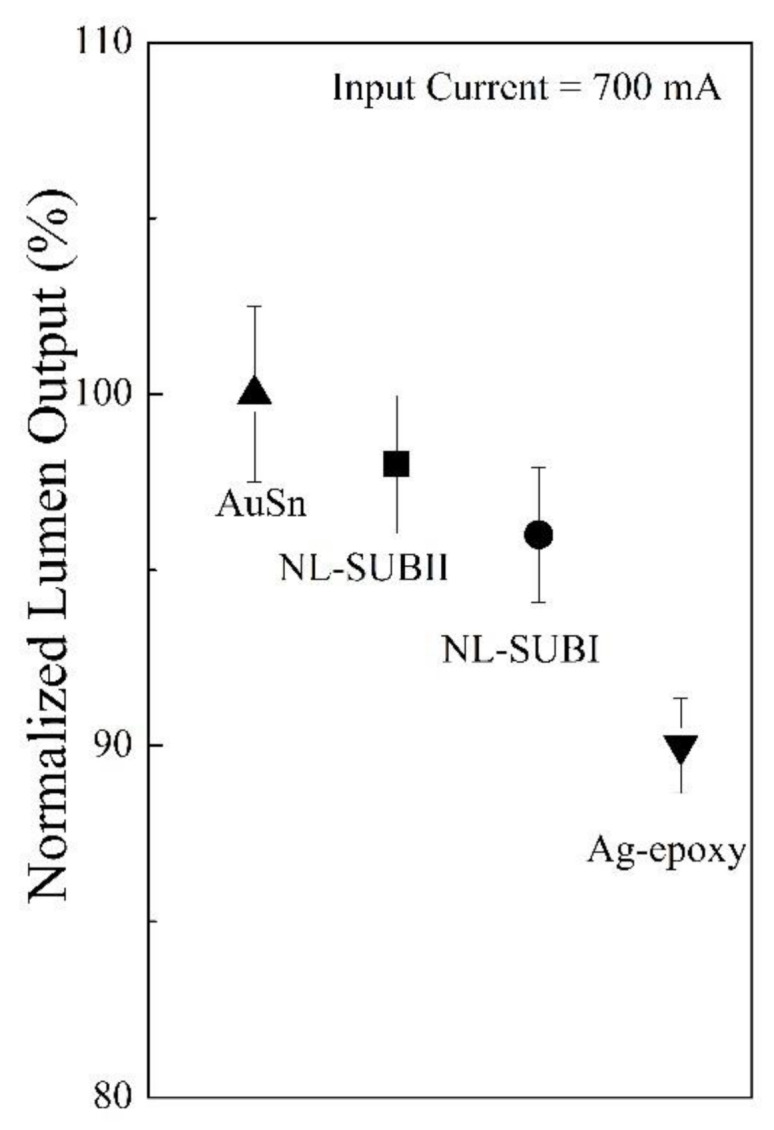
Normalized lumen output of the devices made by the NL bonding method with different substrates and two conventional die–substrate bonding methods at an input current of 700 mA: the solid circle symbol (●) represents NL bonding with substrate type-I; the solid square symbol (■) represents NL bonding with substrate type-II; the solid upside triangle (▲) represents AuSn bonding with an industrial standard BLT value of 20 ± 2 μm; and the solid downside triangle (▼) represents Ag-epoxy bonding with an industrial standard BLT value of 25 ± 2 μm.

Figure 8 presents the optical performance in terms of normalized lumen output at the suggested maximum input current of 700 mA for the devices made by the NL bonding method with different substrates and the comparison to two conventional die–substrate bonding methods. It is obvious that the lumen output for the devices made by NL bonding with package substrate type II (SUB-II) was enhanced about 2% higher compared with the devices using package substrate type I (SUB-I). This is fully consistent with the prior results on the dependence of $T_{j}$ and $R_{th}$ on the total contact number and contact area under the same BLT. In addition, the lumen output for the devices made by the NL bonding approach with package substrate type I (SUB-I) was about 6% higher than the Ag-epoxy bonding, and about 4% lower than AuSn bonding.

### 3.5. Devices Performance: Long-Term Reliability

**Figure 9:**
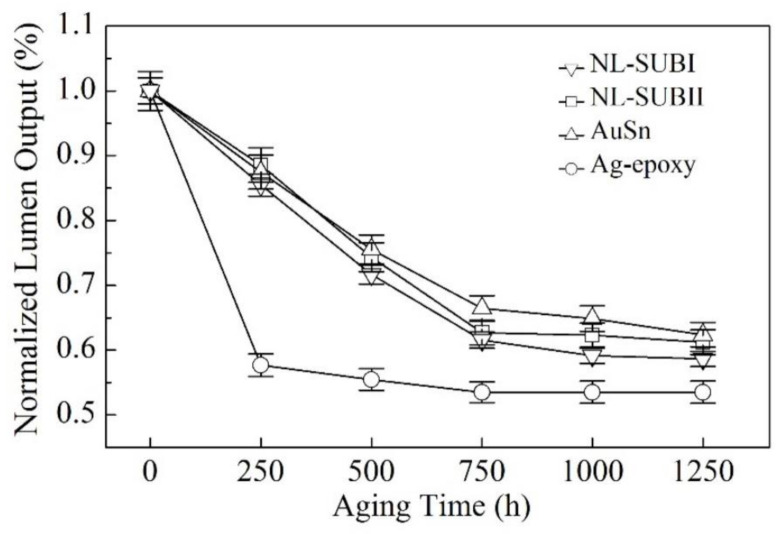
Long-term lumen maintenance of the devices made by the NL bonding method with different substrates and two conventional die-substrate bonding methods as a function of aging time under the stressing condition of an operating current of 700 mA, a relative humidity RH = 85%, and a high environmental temperature of 85 °C: the open downside triangle symbol (▽) represents the experimental data for the device made by NL bonding with substrate type-I; the open square symbol (□) represents the experimental data for the device made by NL bonding with substrate type-II; the open upside triangle symbol (△) represents Ag-epoxy bonding with an industrial standard BLT value of 25 ± 2 μm; and the open circle symbol (◯) represents the experimental data of Ag-epoxy bonding with an industrial standard BLT value of 20 ± 2 μm. This Wet High Temperature Operating Life (WHTOL) test goes beyond the requirement of the standard JEDEC No. 22-A101C while extending the test duration by 25% from 1000 to 1250 h.

Figure 9 presents a comparison of the aging time-dependent lumen maintenance of the devices made with the NL approach with different substrates and the other two conventional methods under the industrial standard condition of high chamber temperature of 85 °C and high relative humidity of 85% for a total duration of 1250 h. The y-axis represents relative change in the lumen maintenance normalized to the initial lumen output. The x-axis represents the aging or stressing time. It is evident that at the aging time of 1250 h, in the devices made by the NL bonding method, the lumen maintenance using package substrate type II (SUB-II) was about 3% higher than the devices using package substrate type I (SUB-I). The lumen maintenance of the packaged LEDs made by NL with packaged substrate type I (SUB-I) was about 4% lower than the device made by AuSn bonding and about 5.1% higher than the device made by the Ag-epoxy bonding. The superior reliability associated with the NL bonding method with package substrate type II (SUB-II) compared with the devices using package substrate type I (SUB-I) evidently resulted from the observed reduced electrical resistance as well as a reduction in thermal resistance, as discussed above.

## 4. Conclusions

An innovative off-chip bonding method was explored and its “nano-locking” concept for die–substrate interconnection and bonding was demonstrated as an example by its application for the attachment of high-power GaN based semiconductor dies to its package substrate. The surface morphology of the bonding surfaces plays an important role in forming the real total number of contacts and overall contact area between the peaks and valleys on the two bonding surfaces. A mathematical model was proposed to describe and predict the electrical (and thermal) conductivity of the overall real contact area between the bonding surfaces. By analyzing the surface height distribution of the metallic pads on the two different package substrates, larger frequency percent of qualified surface roughness height distribution contributed to smaller electrical (and thermal) resistance between the two bonding surfaces, resulting in a lower overall device electrical resistance and a reduced thermal resistance, thus an improved overall electrical, thermal, optical device performance and reliability. The present work opens a new direction for scalable, reliable, and simple nanoscale off-chip electrical interconnection and bonding for nano- and micro-electrical devices as well as other functional devices.

## Figures and Tables

**Table 1:** Commercially Die-substrate Bonding Material Properties.

Bonding Material	Volume Resistivity	Bond-Line Thickness (BLT)
Ag-epoxy (85% by weight)	8 $\times \;10^{-5}\;$Ωm	25 ± 2 μm
AuSn (80% Gold, 20% Tin)	1.64 $\times \;10^{-7}\;$Ωm	20 ± 2 μm

**Table 2:** Frequency percent distribution of surface roughness heights.

Semiconductor Die		Substrate	SUB-I	SUB-II
Heights (nm)	Frequency Percent	Heights (nm)	Frequency Percent	Frequency Percent
0–10	0.2242	90–100	0	0.0113
10–20	0.1529	80–90	0	0.0160
20–30	0.0761	70–80	0	0.0160
30–40	0.0324	60–70	0	0.0222
40–50	0.0138	50–60	0.0016	0.0264
50–60	0.0035	40–50	0.0131	0.0456
60–70	0.0004	30–40	0.096	0.0604
−10–0	0.2248	100–110	0	0.0071
		**Total**	**0.11**	**0.21**

## Data Availability

No new data were created or analyzed in this study. Data sharing is not applicable to this article.
